# Supplementary material for: Fasting blood glucose and risk of incident pancreatic cancer
Source: PLoS One. 2022 Oct 27;17(10):e0274195. doi: 10.1371/journal.pone.0274195 (PMC9612540; doi:10.1371/journal.pone.0274195)
Supplement: S1 Table — (DOC) [file pone.0274195.s001.doc]

**S1 Table. Comparison between participants with and without incident pancreatic cancer.**

| **Characteristic** | **Without incident pancreatic cancer (N=18,669)** | **With incident pancreatic cancer (N=381)** | ***P*-value^*^** |
| --- | --- | --- | --- |
| Age (years) | 64.5 ± (10.1) | 64.7 ± (10.1) | 0.751 |
| Gender |  |  | 0.934 |
| Male (%) | 12,065 (64.6) | 247 (64.8) |  |
| Female (%) | 6,604 (35.4) | 134 (35.2) |  |
| BMI (kg/m^2^) | 23.9 ± (3.0) | 23.9 ± (2.9) | 0.954 |
| Systolic BP (mmHg) | 127.5 ± (15.8) | 127.6 ± (15.6) | 0.952 |
| Diastolic BP (mmHg) | 78.0 ± (9.9) | 77.6 ± (10.2) | 0.446 |
| Total cholesterol (mg/dL) | 195.3 ± (38.0) | 195.0 ± (37.5) | 0.882 |
| Triglyceride (mg/dL) | 143.9 ± (93.9) | 146.7 ± (92.5) | 0.559 |
| HDL-cholesterol (mg/dL) | 54.9 ± (33.8) | 50.9 ± (12.9) | <0.001 |
| LDL-cholesterol (mg/dL) | 113.9 ± (38.3) | 115.6 ± (37.5) | 0.394 |
| Fasting blood glucose (mg/dL) | 102.9 ± (26.9) | 109.8 ± (35.5) | <0.001 |
| SCr (mg/dL) | 1.16 ± (1.40) | 1.19 ± (1.55) | 0.729 |
| eGFR (mL/min per 1.73m^2^) | 76.3 ± (19.9) | 75.9 ± (19.3) | 0.732 |
| AST (U/L) | 28.2 ± (23.2) | 29.2 ± (32.0) | 0.545 |
| ALT (U/L) | 25.7 ± (22.6) | 25.9 ± (17.2) | 0.737 |
| GGT (U/L) | 48.1 ± (84.8) | 51.3 ± (84.9) | 0.450 |
| Smoking amount (pack-year) | 11.6 ± (18.5) | 11.5 ± (20.7) | 0.938 |
| Alcohol intake (%) | 18.7 | 19.0 | 0.888 |
| Physical activity (%) | 13.0 | 12.3 | 0.686 |

Data are expressed as means (standard deviation) or percentages.

^*^*P*-value by t-test for continuous variables and Chi square test for categorical variables.
