# Supplementary material for: Fasting blood glucose and risk of incident pancreatic cancer
Source: PLoS One. 2022 Oct 27;17(10):e0274195. doi: 10.1371/journal.pone.0274195 (PMC9612540; doi:10.1371/journal.pone.0274195)
Supplement: S2 Table — (DOC) [file pone.0274195.s002.doc]

**S2 Table. Hazard ratios (HRs) and 95% confidence intervals (CI) for the incidence of pancreatic cancer according to the categories of fasting blood glucose in model that including triglyceride** as a covariate.

| \ | HR (95% CI) ^*^ | |
| --- | --- | --- |
|  | Unadjusted | Multivariate adjusted model |
| **Fasting blood glucose levels** |  |  |
| Quartile 1 | 1.00 (reference) | 1.00 (reference) |
| Quartile 2 | 1.39 (1.01-1.92) | 1.45 (1.01-2.08) |
| Quartile 3 | 1.50 (1.09-2.07) | 1.55 (1.08-2.23) |
| Quartile 4 | 2.18 (1.62-2.95) | 2.37 (1.72-3.27) |
| *P* for trend | <0.001 | <0.001 |
| Age |  | 0.996 (0.985-1.008) |
| Gender (female vs male) |  | 0.964 (0.752-1.237) |
| BMI |  | 0.981 (0.947-1.017) |
| Systolic BP |  | 0.999 (0.992-1.006) |
| Triglyceride |  | 1.000 (0.999-1.001) |
| GGT |  | 1.000 (0.999-1.001) |
| eGFR |  | 1.001 (0.995-1.006) |
| Smoking amount (pack-year) |  | 0.999 (0.993-1.006) |
| Alcohol intake |  | 0.974 (0.730-1.299) |
| Physical activity |  | 0.946 (0.694-1.291) |

Multivariate adjusted model was adjusted for age, gender, BMI, systolic BP, triglyceride, GGT, eGFR, smoking amount (pack-year), alcohol intake and physical activity.
