# Supplementary material for: Fasting blood glucose and risk of incident pancreatic cancer
Source: PLoS One. 2022 Oct 27;17(10):e0274195. doi: 10.1371/journal.pone.0274195 (PMC9612540; doi:10.1371/journal.pone.0274195)
Supplement: S3 Table — (DOC) [file pone.0274195.s003.doc]

**S3 Table. Hazard ratios (HRs) and 95% confidence intervals (CI) for the incidence of pancreatic cancer according to the categories of fasting blood glucose**

|  | HR (95% CI) ^*^ | |
| --- | --- | --- |
|  | Unadjusted | Multivariate adjusted |
| **Categories of fasting blood glucose** |  |  |
| Normal | 1.00 (reference) | 1.00 (reference) |
| Impaired fasting glucose | 1.30 (1.01-1.69) | 1.32 (1.01-1.72) |
| Diabetes mellitus | 2.00 (1.59 -2.52) | 2.06 (1.61-2.62) |
| *P* for trend | <0.001 | <0.001 |
| Age (years) |  | 0.996 (0.984-1.007) |
| Gender (female vs male) |  | 0.961 (0.746-1.238) |
| BMI |  | 0.982 (0.948-1.017) |
| Systolic BP |  | 0.999 (0.993-1.006) |
| Total cholesterol |  | 1.000 (0.997-1.003) |
| GGT |  | 1.000 (0.999-1.001) |
| eGFR |  | 1.000 (0.995-1.006) |
| Smoking amount (pack-year) |  | 0.999 (0.993-1.005) |
| Alcohol intake |  | 0.996 (0.747-1.329) |
| Physical activity |  | 0.947 (0.694-1.291) |

Multivariate adjusted model was adjusted for age, gender, BMI, systolic BP, total cholesterol, GGT, eGFR, smoking amount (pack-year), alcohol intake and physical activity.

Normal: fasting glucose < 100 mg/dL, impaired fasting glucose: fasting glucose 100-125 mg/dL and diabetes mellitus: fasting glucose ≥ 126 mg/dL or presence of DM.
