# Supplementary material for: Fasting blood glucose and risk of incident pancreatic cancer
Source: PLoS One. 2022 Oct 27;17(10):e0274195. doi: 10.1371/journal.pone.0274195 (PMC9612540; doi:10.1371/journal.pone.0274195)
Supplement: S4 Table — (DOC) [file pone.0274195.s004.doc]

**S4 Table. Hazard ratios (HRs) and 95% confidence intervals (CI) for the incidence of pancreatic cancer according to the quartile of fasting blood glucose after excluding the possibility of 6-months reverse causality (N=19,028)**

|  | HR (95% CI) ^*^ | |
| --- | --- | --- |
|  | Unadjusted | Multivariate adjusted model |
| **Fasting blood glucose levels** |  |  |
| Quartile 1 | 1.00 (reference) | 1.00 (reference) |
| Quartile 2 | 1.31 (0.94-1.81) | 1.37 (0.98-1.91) |
| Quartile 3 | 1.41 (1.02-1.95) | 1.51 (1.08-2.11) |
| Quartile 4 | 2.02 (1.49-2.74) | 2.14 (1.55-2.95) |
| *P* for trend | <0.001 | <0.001 |
| Age |  | 0.998 (0.987-1.010) |
| Gender (female vs male) |  | 1.009 (0.778-1.310) |
| BMI |  | 0.979 (0.944-1.016) |
| Systolic BP |  | 0.999 (0.992-1.006) |
| Total cholesterol |  | 0.999 (0.996-1.002) |
| GGT |  | 1.000 (0.998-1.001) |
| eGFR |  | 1.000 (0.994-1.005) |
| Smoking amount (pack-year) |  | 1.000 (0.993-1.007) |
| Alcohol intake |  | 0.968 (0.718-1.305) |
| Physical activity |  | 0.944 (0.685-1.301) |

359 incident cases of pancreatic cancer developed between 2009 and 2013 after excluding the possibility of 6-months reverse causality.

Multivariate adjusted model was adjusted for age, gender, BMI, systolic BP, total cholesterol, GGT, eGFR, smoking amount (pack-year), alcohol intake and physical activity.
