# Supplementary material for: Fasting blood glucose and risk of incident pancreatic cancer
Source: PLoS One. 2022 Oct 27;17(10):e0274195. doi: 10.1371/journal.pone.0274195 (PMC9612540; doi:10.1371/journal.pone.0274195)
Supplement: S5 Table — (DOC) [file pone.0274195.s005.doc]

**S5 Table. Hazard ratios (HRs) and 95% confidence intervals (CI) for the incidence of pancreatic cancer according to the quartile of fasting blood glucose after** **excluding the possibility of 1-year reverse causality (N=18,995)**

|  | HR (95% CI) ^*^ | |
| --- | --- | --- |
|  | Unadjusted | Multivariate adjusted model |
| **Fasting blood glucose levels** |  |  |
| Quartile 1 | 1.00 (reference) | 1.00 (reference) |
| Quartile 2 | 1.33 (0.95-1.87) | 1.40 (0.99-1.98) |
| Quartile 3 | 1.36 (0.96-1.90) | 1.47 (1.04-2.09) |
| Quartile 4 | 1.97 (1.43-2.70) | 2.08 (1.49-2.92) |
| *P* for trend | <0.001 | <0.001 |
| Age |  | 1.000 (0.988-1.012) |
| Gender (female vs male) |  | 1.013 (0.771-1.330) |
| BMI |  | 0.985 (0.949-1.024) |
| Systolic BP |  | 0.999 (0.992-1.006) |
| Total cholesterol |  | 0.999 (0.996-1.002) |
| GGT |  | 1.000 (0.999-1.001) |
| eGFR |  | 1.000 (0.994-1.006) |
| Smoking amount (pack-year) |  | 0.999 (0.993-1.006) |
| Alcohol intake |  | 0.956 (0.698-1.311) |
| Physical activity |  | 0.970 (0.695-1.354) |

326 incident cases of pancreatic cancer developed between 2009 and 2013 after excluding the possibility of 1-year reverse causality.

Multivariate adjusted model was adjusted for age, gender, BMI, systolic BP, total cholesterol, GGT, eGFR, smoking amount (pack-year), alcohol intake and physical activity.
