# Supplementary figures and images for: Fasting blood glucose and risk of incident pancreatic cancer
Source: PLoS One. 2022 Oct 27;17(10):e0274195. doi: 10.1371/journal.pone.0274195 (PMC9612540; doi:10.1371/journal.pone.0274195)

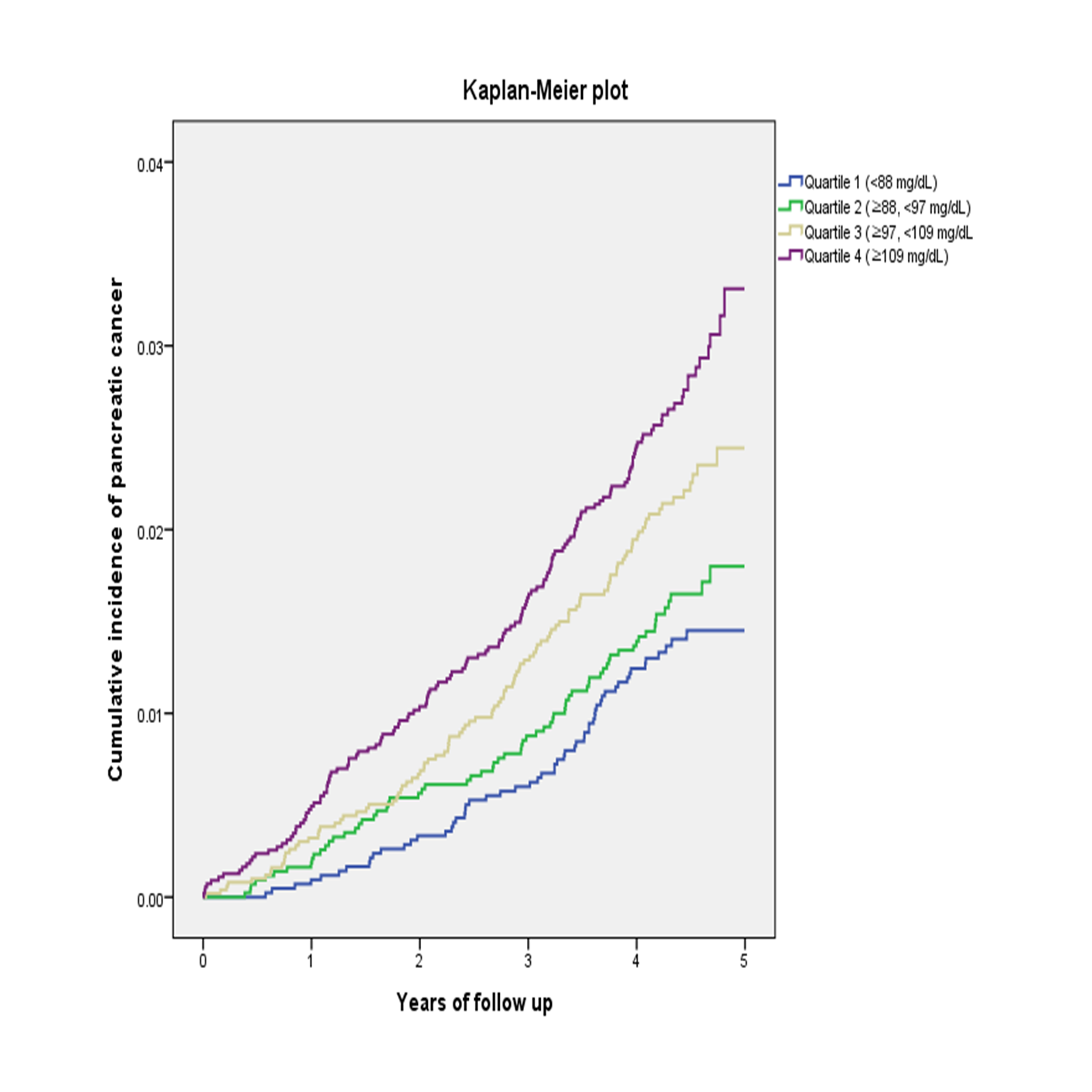

Supplement: S1 Fig — (TIF) [file pone.0274195.s006.tif]
