# Supplementary material for: Fasting blood glucose and risk of incident pancreatic cancer
Source: PLoS One. 2022 Oct 27;17(10):e0274195. doi: 10.1371/journal.pone.0274195 (PMC9612540; doi:10.1371/journal.pone.0274195)
Supplement: Attachment — Submitted filename: Response to reviewers and editors.docx [file pone.0274195.s007.docx]

**Response to reviewers and editors’ request**

**TITLE: Fasting blood glucose and risk of incident pancreatic cancer**

Reviewer(s)' Comments:

Reviewer #1: In this article, Kim et al examined the association of fasting blood glucose level and incident pancreatic cancer risk using Korea National health Information Database. The study included 19,050 participants at the baseline. 381 incident cases were observed during an average of 4.32 years follow-up. The authors found the increase of blood glucose level was associated with pancreatic cancer in each quartile, even in the pre diabetic range.

There are several concerns of the study.
1. The authors indicated that the dataset was sampled and represented a portion of Korea National health Information Database. It would be useful to describe how the sampling was done and if it is representative of the population.

🡪 We are very appreciating reviewer #1’s point. As the suggestion by the reviewers, the lower sentence is a newly added sentence actually described in the materials and method section.

Line 129-131: “This sampled database was constructed by systematic stratified random sampling allocated proportionally within each class of age, sex, income level and eligibility status of medical insurance.”

Reference) Lee J, Lee JS, Park SH, Shin SA, Kim K. Cohort Profile: The National Health Insurance Service-National Sample Cohort (NHIS-NSC), South Korea. Int J Epidemiol. 2017;46(2):e15.

2. It is unclear why authors chose the case-control study design with 1:50 matching controls. Why not the entire dataset? Also In page 13, Result section, “During 82,276.8 person-years of follow-up, 381 (2.00 %) incident cases of pancreatic cancer developed between 2009 and 2013.” This is misleading because the authors chose 1:50 matching, but not 2% of incident rate.

🡪 We are very appreciating reviewer #1’s point. In the initial analysis, we observed 223,298 people from 2009 to 2013, and the incidence cases of pancreatic cancer was 381 (0.17%), which was very rare. Therefore, we applied matched study design to increase the statistical efficiency.

The lower sentences are the corrected sentence that was actually described in revised manuscript.

Line 143-145: “Only 381 incident cases of pancreatic cancer developed between 2009 and 2013. Therefore, we adopted matched design to increase the statistical efficiency.”

Line 224-225: “During follow-up period, 381 incident cases of pancreatic cancer developed between 2009 and 2013.”

3. It is surprising to see the multivariate adjusted models had much higher HR than the unadjusted models, although the samples were matched for the covariates.

🡪 We are very appreciating reviewer #1’s point. As reviewer 1’s point, generally, the hazard ratio after adjustment is less than the hazard ratio before adjustment. However, our results are not. We believe that using a matched study design contributed to these outcomes. In fact, the significance of confounding variables has disappeared in the multivariate adjust model (Table 2).

4. Quartiles of glucose levels were examined in the study. It would be interesting to see the group with >126 mg/dL. It would be also interesting to examine if there is any linear association between glucose level and pancreatic cancer.

🡪 We are very appreciating reviewer #1’s point. We analyzed the adjusted hazard ratio of incident pancreatic cancer according to the three groups of fasting blood glucose level (<100 mg/dL, 100-125 mg/dL, ≥126 mg/dL or presence of DM). Results of analysis are presented in the supplementary table S2. In adjusted model, the hazard ratios and 95% confidence interval for incident pancreatic cancer were 1.32 (95% CI: 1.01-1.72) in the impaired fasting glucose group and 2.06 (95% CI: 1.61-2.62) in the diabetes mellitus group.

**Table S2. Hazard ratios (HRs) and 95% confidence intervals (CI) for the incidence of pancreatic cancer according to the categories of fasting blood glucose**

|  | HR (95% CI) ^*^ | |
| --- | --- | --- |
|  | Unadjusted | Multivariate adjusted |
| **Categories of fasting blood glucose** |  |  |
| Normal | 1.00 (reference) | 1.00 (reference) |
| Impaired fasting glucose | 1.30 (1.01-1.69) | 1.32 (1.01-1.72) |
| Diabetes mellitus | 2.00 (1.59 -2.52) | 2.06 (1.61-2.62) |
| *P* for trend | <0.001 | <0.001 |
| Age (years) |  | 0.996 (0.984-1.007) |
| Gender (female vs male) |  | 0.961 (0.746-1.238) |
| BMI |  | 0.982 (0.948-1.017) |
| Systolic BP |  | 0.999 (0.993-1.006) |
| Total cholesterol |  | 1.000 (0.997-1.003) |
| GGT |  | 1.000 (0.999-1.001) |
| eGFR |  | 1.000 (0.995-1.006) |
| Smoking amount (pack-year) |  | 0.999 (0.993-1.005) |
| Alcohol intake |  | 0.996 (0.747-1.329) |
| Physical activity |  | 0.947 (0.694-1.291) |

Multivariate adjusted model was adjusted for age, gender, BMI, systolic BP, total cholesterol, GGT, eGFR, smoking amount (pack-year), alcohol intake and physical activity.

Normal: fasting glucose < 100 mg/dL, impaired fasting glucose: fasting glucose 100-125 mg/dL and diabetes mellitus: fasting glucose ≥ 126 mg/dL or presence of DM.

Furthermore, there was positive linear association between fasting glucose level and incidence of pancreatic cancer. Fasting glucose level was associated with an unadjusted hazard ratio of 1.007 (95% CI: 1.004-1.009) and an adjusted hazard ratio of 1.007 (95% CI: 1.004-1.010) for the incidence of pancreatic cancer.

5. Does the study account for the effect of diabetic medication?

🡪 We are very appreciating reviewer 1’s point. We categorized the participants into quartile according to their fasting glucose level, regardless of whether they take diabetic medication or not. Therefore, we analyzed how many of the participants were diagnosed with diabetes, and reflected this in the manuscript. The identification of history of diabetes mellitus was based on reviewing data for ICD code of DM (E10-E14).

The lower sentence and table 1 are actually presented in revised manuscript.

Line 179-182: The identification of present baseline DM was based on reviewing data for International Classification of Diseases, 10th Revision, Clinical Modification (ICD-10-CM) code of DM (E10-E14) from 2002 to 2009 years (date of receiving medical health check-up in 2009).

**Table 1. Baseline characteristics of participants according to the quartile groups of fasting blood glucose levels (N=19,050 )**

| Characteristic | Overall | **Fasting blood glucose level** | | | | |
| --- | --- | --- | --- | --- | --- | --- |
|  |  | Quartile 1(<88, n=4,726) | Quartile 2(≥88, <97, n=4,961) | Quartile 3(≥97, <109, n=4,709) | Quartile 4(≥109, n=4,654) | *P*-for trend^*^ |
| Person-year (total) | 82,276.8 | 20,541.9 | 21,528.3 | 20,417.2 | 19,789.4 |  |
| Person-year (average) | 4.32 ± (0.69) | 4.35 ± (0.65) | 4.34 ± (0.65) | 4.33 ± (0.65) | 4.25 ± (0.79) | <0.001 |
| Age (years) | 64.5 ± (10.1) | 64.0 ± (10.5) | 64.1 ± (10.3) | 64.5 ± (10.1) | 65.5 ± (9.6) | <0.001 |
| Gender |  |  |  |  |  | <0.001 |
| Male (%) | 12,312 (64.6) | 2,823 (59.7) | 3,024 (60.9) | 3,153 (67.0) | 3,312 (71.2) |  |
| Female (%) | 6,738 (35.4) | 1,903 (40.3) | 1,937 (39.1) | 1,556 (33.0) | 1,342 (28.8) |  |
| BMI (kg/m^2^) | 23.9 ± (3.0) | 23.5 ± (2.9) | 23.8 ± (2.9) | 24.1 ± (3.0) | 24.5 ± (3.2) | <0.001 |
| Systolic BP (mmHg) | 127.5 ± (15.8) | 125.2 ± (15.6) | 126.5 ± (15.5) | 127.9 ± (15.7) | 130.4 ± (15.9) | <0.001 |
| Diastolic BP (mmHg) | 78.0 ± (9.9) | 76.7 ± (9.8) | 77.6 ± (9.8) | 78.5 ± (9.9) | 79.3 ± (10.0) | <0.001 |
| Total cholesterol (mg/dL) | 195.3 ± (38.0) | 192.4 ± (36.9) | 195.7 ± (36.7) | 197.9 ± (38.0) | 195.2 ± (40.2) | <0.001 |
| Triglyceride (mg/dL) | 120 (85-172) | 109.5 (79-153) | 115 (83-163) | 123 (83-176) | 137 (97-201) | <0.001 |
| HDL-cholesterol (mg/dL) | 54.0 ± (33.5) | 54.7 ± (29.3) | 56.0 ± (38.1) | 55.5 ± (35.4) | 52.9 ± (30.3) | 0.012 |
| LDL-cholesterol (mg/dL) | 113.9 ± (38.3) | 113.7 ± (38.0) | 115.4 ± (37.7) | 115.9 ± (38.1) | 110.7 ± (39.2) | 0.001 |
| SCr (mg/dL) | 1.16 ± (1.39) | 1.10 ± (1.27) | 1.15 ± (1.42) | 1.20 ± (1.49) | 1.20 ± (1.40) | <0.001 |
| eGFR (mL/min per 1.73m^2^) | 76.3 ± (19.9) | 78.0 ± (19.7) | 76.9 ± (19.5) | 75.8 ± (20.2) | 74.5 ± (20.1) | <0.001 |
| AST (U/L) | 24 (20-30) | 24 (20-29) | 24 (20-29) | 24 (20-30) | 25 (20-33) | <0.001 |
| ALT (U/L) | 21 (16-29) | 19 (15-26) | 20 (15-27) | 21 (16-29) | 24 (17-34) | <0.001 |
| GGT (U/L) | 26 (18-45) | 23 (16-37) | 24 (17-39) | 27 (18-46) | 33 (21-60) | <0.001 |
| Smoking amount (pack-year) | 11.6 ± (18.6) | 10.6 ± (17.8) | 10.2± (17.3) | 12.0 ± (18.9) | 13.8 ± (20.2) | <0.001 |
| Alcohol intake (%) | 18.7 | 14.3 | 16.2 | 20.3 | 24.2 | <0.001 |
| Physical activity (%) | 13.0 | 11.7 | 13.2 | 13.3 | 14.0 | <0.001 |
| Presence of DM (%) | 3914 (20.6) | 513 (10.8) | 564 (11.4) | 751 (15.9) | 2086 (44.8) | <0.001 |
| Development of pancreatic cancer (%) | 381 (2.00) | 63 (1.33) | 92 (1.85) | 94 (2.00) | 132 (2.84) | <0.001 |

Data are means (standard deviation), medians (interquartile range), or percentages.

^*^*P*-value by ANOVA-test for continuous variables and Chi square test for categorical variables.

6. In the results section, it is unclear “As expected, all variables were statistically insignificant except HDL-cholesterol and fasting blood glucose, as it was the result after matching”. Does it mean that they are not significant between cases and controls? If so, please indicate it.

🡪 We are very appreciating reviewer 1’s point. Because we adopted matched study design, there is no difference in variables except HDL-cholesterol and fasting blood glucose between the control group and the cases. In order to convey our meaning clearly, we revised it to the following sentence

Line 229-232: As it was the result after matching, there was no statistically significant difference in all variables except HDL-cholesterol and fasting blood glucose between participant who did not develop pancreatic cancer and patients with pancreatic cancer (Supplementary Table S1).

7. Please provide a Kaplan-Meier plot to show the incident rate for different quartiles

🡪 We are very appreciating reviewer 1’s point. As review 1’s suggestion, the figure below is Kaplan-Meier plot of incidence of pancreatic cancer according to quartiles.

Figure. Cumulative incidence for the development of pancreatic cancer

8. Proofreading would be needed to correct typos and grammatical errors.

🡪 We are very appreciating reviewer 1’s point. Typos and grammatical errors were corrected through correction.

Reviewer #2: Authors are carried out retrospective analysis of patient visits to the fasting glucose levels vs pancreatic cancer incident rates. Overall authors used large pool of Korean patients with fasting glucose levels ranging <88 to >109 and patients with higher quartiles of fasting glucose significantly associated with pancreatic incidence.

Minor Comments.

1. Authors should comment on quartile 4 have a higher triglycerides - thus any effect on the pancreatic cancer incidence as independent of fasting glucose levels?

🡪 We are very appreciating to reviewer #2’s point. The table below shows the hazard ratio of the incidence of pancreatic cancer in model that including triglyceride as a covariate.

Table. Hazard ratios (HRs) and 95% confidence intervals (CI) for the incidence of pancreatic cancer according to the categories of fasting blood glucose

|  | HR (95% CI) ^*^ | |
| --- | --- | --- |
|  | Unadjusted | Multivariate adjusted model |
| **Fasting blood glucose levels** |  |  |
| Quartile 1 | 1.00 (reference) | 1.00 (reference) |
| Quartile 2 | 1.39 (1.01-1.92) | 1.45 (1.01-2.08) |
| Quartile 3 | 1.50 (1.09-2.07) | 1.55 (1.08-2.23) |
| Quartile 4 | 2.18 (1.62-2.95) | 2.37 (1.72-3.27) |
| *P* for trend | <0.001 | <0.001 |
| Age |  | 0.996 (0.985-1.008) |
| Gender (female vs male) |  | 0.964 (0.752-1.237) |
| BMI |  | 0.981 (0.947-1.017) |
| Systolic BP |  | 0.999 (0.992-1.006) |
| Triglyceride |  | 1.000 (0.999-1.001) |
| GGT |  | 1.000 (0.999-1.001) |
| eGFR |  | 1.001 (0.995-1.006) |
| Smoking amount (pack-year) |  | 0.999 (0.993-1.006) |
| Alcohol intake |  | 0.974 (0.730-1.299) |
| Physical activity |  | 0.946 (0.694-1.291) |

Multivariate adjusted model was adjusted for age, gender, BMI, systolic BP, triglyceride, GGT, eGFR, smoking amount (pack-year), alcohol intake and physical activity.

2. Is their any difference in BMI of Male vs Female?
🡪 We are very appreciating to reviewer #2’s point. The average BMI for men (12,310 people) and women (6,733 people) among the participants is 23.8 (±2.9) kg/m^2^ and 24.2(±3.2) kg/m^2^, respectively. As a result of the t test, there was a statistically significant difference (*P*<0.01).

3. Smoking is higher in the Quartile 4- does authors analyzed any effect on the pancreatic cancer

🡪 We are very appreciating to reviewer #2’s point. In adjusted model, the hazard ratio of incidence of pancreatic cancer to smoking status is 1.000 (0.993-1.006) (Table 2).

Reviewer #3: Early detection of pancreatic cancer is challenging due to clinically silent progression of the disease until it reaches an advanced stage. It is important to understand and identify modifiable risk factors as it can help develop strategies to mitigate the risk of cancer. There are some known lifestyle-related risk factors such as alcohol and tobacco smoking, and metabolic syndrome such as obesity and diabetes mellitus (DM). The purpose of the submitted retrospective cohort study was to analyze the risk of pancreatic cancer according to the baseline level of fasting glucose in the South Korean population for which they used the National Health Insurance Corporation (NHIC) database that covers 97% of the population. They obtained data from 223,551 medical checkup participants included in NHIC database in 2009 and excluded those with previous pancreatic cancer diagnosis or with no fasting glucose data. The analysis included a total of 381 incident cases of pancreatic cancer that developed between 2009 and 2013, and 18,669 controls that were randomly selected from the participants after 1:50 matching for covariates resulting in a total of 19,050 participants. The participants were analyzed as quartile groups according to the fasting blood glucose level at the time of enrollment (quartile 1: <88 mg/dL, quartile 2: 88−97 mg/dL, quartile 3: 97−109 mg/dL and quartile 4: ≥109 mg/dL), and multivariate Cox proportional hazards models were used to estimate adjusted HRs and 95% CIs for incident pancreatic cancer. The results showed that that the risk of pancreatic cancer increased statistically significantly with fasting blood glucose level (quartile 1 [reference] vs. quartiles 2, 3 and 4) with prediabetic ranges, even after adjusting for multiple covariates. The authors mentioned that this study adds value to the existing literature by providing a clearer correlation between fasting blood glucose levels and pancreatic cancer incidence.

Several studies have already been conducted in Korea and elsewhere reporting the association of not only DM but also of elevated fasting blood glucose with increased risk of pancreatic cancer. A 2019 study by Koo et al. (reference 5) used the same South Korean national database as the current manuscript to report HRs for fasting glucose level and pancreatic cancer incidence. They reported a linear relationship between fasting glucose levels and pancreatic cancer incidence where the cumulative incidence rate of pancreatic cancer increased statistically significantly with elevation in fasting glucose level, not only in those with DM but also in those with prediabetes or high normal range of fasting blood glucose levels, and even after adjustment of well-known risk factors. Therefore, the reviewer’s enthusiasm has been substantially lowered by the lack of novelty in the submitted manuscript. In addition, this reviewer also thought the study could have explored areas that other studies based on the Korean population may not have explored, for example, include data from a longer period and analyze longitudinal trajectory of fasting glucose levels, assuming that longitudinal glucose measurements are available in the database as the population undergoes check up every two years, as this could have added a new dimension to the analysis. The reason for quartile 4 threshold is not clear, and why the quartiles are not distinct for ≥109 − <126 mg/dL and ≥126 mg/dL? The reviewer was unable to find any information on anti-diabetes medication and the multivariate analysis accounted for age, gender, BMI, systolic BP, total cholesterol, GGT, eGFR, smoking amount (pack-year), alcohol intake and physical activity. Furthermore, many patients may have had occult/subclinical pancreatic cancer where reverse causality could be the reason for increased glucose level and although it was not possible to know that in this retrospective setting, an assessment of the duration of hyperglycemia and longitudinal data on glucose levels would have been useful. Thanks to the authors for acknowledging some limitations of their study. This reviewer thinks HbA1c levels were not available in the NHIC database, which is why the study was unable to include the data. The manuscript is well-written overall and easy to read.

🡪 We are very appreciating to reviewer #3’s point. As reviewer #3’ pointed out, previous study has already reported the relationship between fasting blood glucose levels and the incidence of pancreatic cancer (Koo et al, reference no.5). Although, we believe our study contributes to revealing clearer relationship between fasting blood glucose levels and the incidence of pancreatic cancer at pre-diabetic range. Following reviewer #3's suggestion, we conducted further research as follows.

First of all, we analyzed the adjusted hazard ratio of the incident pancreatic cancer according to the three groups of fasting blood glucose level (<100 mg/dL, 100-125 mg/dL, ≥126 mg/dL or presence of DM). Results of analysis are presented in the supplementary table S2. In adjusted model, the hazard ratios and 95% confidence interval for incident pancreatic cancer were 1.32 (95% CI: 1.01-1.72) in the impaired fasting glucose group and 2.06 (95% CI: 1.61-2.62) in the diabetes mellitus group.

**Table S2. Hazard ratios (HRs) and 95% confidence intervals (CI) for the incidence of pancreatic cancer according to the categories of fasting blood glucose**

|  | HR (95% CI) ^*^ | |
| --- | --- | --- |
|  | Unadjusted | Multivariate adjusted |
| **Categories of fasting blood glucose** |  |  |
| Normal | 1.00 (reference) | 1.00 (reference) |
| Impaired fasting glucose | 1.30 (1.01-1.69) | 1.32 (1.01-1.72) |
| Diabetes mellitus | 2.00 (1.59 -2.52) | 2.06 (1.61-2.62) |
| *P* for trend | <0.001 | <0.001 |
| Age (years) |  | 0.996 (0.984-1.007) |
| Gender (female vs male) |  | 0.961 (0.746-1.238) |
| BMI |  | 0.982 (0.948-1.017) |
| Systolic BP |  | 0.999 (0.993-1.006) |
| Total cholesterol |  | 1.000 (0.997-1.003) |
| GGT |  | 1.000 (0.999-1.001) |
| eGFR |  | 1.000 (0.995-1.006) |
| Smoking amount (pack-year) |  | 0.999 (0.993-1.005) |
| Alcohol intake |  | 0.996 (0.747-1.329) |
| Physical activity |  | 0.947 (0.694-1.291) |

Multivariate adjusted model was adjusted for age, gender, BMI, systolic BP, total cholesterol, GGT, eGFR, smoking amount (pack-year), alcohol intake and physical activity.

Normal: fasting glucose < 100 mg/dL, impaired fasting glucose: fasting glucose 100-125 mg/dL and diabetes mellitus: fasting glucose ≥ 126 mg/dL or presence of DM.

Second, we categorized the participants into quartile according to their fasting glucose level, regardless of whether they take diabetic medication or not. Therefore, we analyzed how many of the participants were diagnosed with diabetes, and reflected this in the manuscript. The identification of history of diabetes mellitus was based on reviewing data for ICD code of DM (E10-E14).

The lower sentence is a newly added sentence actually described in the materials and method section.

Line 179-182: The identification of present baseline DM was based on reviewing data for International Classification of Diseases, 10th Revision, Clinical Modification (ICD-10-CM) code of DM (E10-E14) from 2002 to 2009 years (date of receiving medical health check-up in 2009).

The table 1 below is a corrected table actually contain information about presence of DM presented in the result section.

**Table 1. Baseline characteristics of participants according to the quartile groups of fasting blood glucose levels (N=19,050 )**

| Characteristic | Overall | **Fasting blood glucose level** | | | | |
| --- | --- | --- | --- | --- | --- | --- |
|  |  | Quartile 1(<88, n=4,726) | Quartile 2(≥88, <97, n=4,961) | Quartile 3(≥97, <109, n=4,709) | Quartile 4(≥109, n=4,654) | *P*-for trend^*^ |
| Person-year (total) | 82,276.8 | 20,541.9 | 21,528.3 | 20,417.2 | 19,789.4 |  |
| Person-year (average) | 4.32 ± (0.69) | 4.35 ± (0.65) | 4.34 ± (0.65) | 4.33 ± (0.65) | 4.25 ± (0.79) | <0.001 |
| Age (years) | 64.5 ± (10.1) | 64.0 ± (10.5) | 64.1 ± (10.3) | 64.5 ± (10.1) | 65.5 ± (9.6) | <0.001 |
| Gender |  |  |  |  |  | <0.001 |
| Male (%) | 12,312 (64.6) | 2,823 (59.7) | 3,024 (60.9) | 3,153 (67.0) | 3,312 (71.2) |  |
| Female (%) | 6,738 (35.4) | 1,903 (40.3) | 1,937 (39.1) | 1,556 (33.0) | 1,342 (28.8) |  |
| BMI (kg/m^2^) | 23.9 ± (3.0) | 23.5 ± (2.9) | 23.8 ± (2.9) | 24.1 ± (3.0) | 24.5 ± (3.2) | <0.001 |
| Systolic BP (mmHg) | 127.5 ± (15.8) | 125.2 ± (15.6) | 126.5 ± (15.5) | 127.9 ± (15.7) | 130.4 ± (15.9) | <0.001 |
| Diastolic BP (mmHg) | 78.0 ± (9.9) | 76.7 ± (9.8) | 77.6 ± (9.8) | 78.5 ± (9.9) | 79.3 ± (10.0) | <0.001 |
| Total cholesterol (mg/dL) | 195.3 ± (38.0) | 192.4 ± (36.9) | 195.7 ± (36.7) | 197.9 ± (38.0) | 195.2 ± (40.2) | <0.001 |
| Triglyceride (mg/dL) | 120 (85-172) | 109.5 (79-153) | 115 (83-163) | 123 (83-176) | 137 (97-201) | <0.001 |
| HDL-cholesterol (mg/dL) | 54.0 ± (33.5) | 54.7 ± (29.3) | 56.0 ± (38.1) | 55.5 ± (35.4) | 52.9 ± (30.3) | 0.012 |
| LDL-cholesterol (mg/dL) | 113.9 ± (38.3) | 113.7 ± (38.0) | 115.4 ± (37.7) | 115.9 ± (38.1) | 110.7 ± (39.2) | 0.001 |
| SCr (mg/dL) | 1.16 ± (1.39) | 1.10 ± (1.27) | 1.15 ± (1.42) | 1.20 ± (1.49) | 1.20 ± (1.40) | <0.001 |
| eGFR (mL/min per 1.73m^2^) | 76.3 ± (19.9) | 78.0 ± (19.7) | 76.9 ± (19.5) | 75.8 ± (20.2) | 74.5 ± (20.1) | <0.001 |
| AST (U/L) | 24 (20-30) | 24 (20-29) | 24 (20-29) | 24 (20-30) | 25 (20-33) | <0.001 |
| ALT (U/L) | 21 (16-29) | 19 (15-26) | 20 (15-27) | 21 (16-29) | 24 (17-34) | <0.001 |
| GGT (U/L) | 26 (18-45) | 23 (16-37) | 24 (17-39) | 27 (18-46) | 33 (21-60) | <0.001 |
| Smoking amount (pack-year) | 11.6 ± (18.6) | 10.6 ± (17.8) | 10.2± (17.3) | 12.0 ± (18.9) | 13.8 ± (20.2) | <0.001 |
| Alcohol intake (%) | 18.7 | 14.3 | 16.2 | 20.3 | 24.2 | <0.001 |
| Physical activity (%) | 13.0 | 11.7 | 13.2 | 13.3 | 14.0 | <0.001 |
| Presence of DM (%) | 3914 (20.6) | 513 (10.8) | 564 (11.4) | 751 (15.9) | 2086 (44.8) | <0.001 |
| Development of pancreatic cancer (%) | 381 (2.00) | 63 (1.33) | 92 (1.85) | 94 (2.00) | 132 (2.84) | <0.001 |

Data are means (standard deviation), medians (interquartile range), or percentages.

^*^*P*-value by ANOVA-test for continuous variables and Chi square test for categorical variables.

Third, as the suggestion by the reviewer, we did further analysis to minimize reverse causality. We calculated hazard ration of the incident pancreatic cancer after excluding participants who developed pancreatic cancer within the 6 month and 1 year of cohort entry.

The lower sentence is a newly added sentence actually described in the materials and method section.

Line 210-212: To minimize potential reverse causality, we conducted additional analysis with excluding participants who developed pancreatic cancer within six months or one year of cohort entry, respectively. The results were presented in supplementary tables.

The supplementary tables below are a newly added tables actually presented in the supplementary file.

**Table S3. Hazard ratios (HRs) and 95% confidence intervals (CI) for the incidence of pancreatic cancer according to the quartile of fasting blood glucose after excluding the possibility of 6-months reverse causality (N=19,028)**

|  | HR (95% CI) ^*^ | |
| --- | --- | --- |
|  | Unadjusted | Multivariate adjusted model |
| **Fasting blood glucose levels** |  |  |
| Quartile 1 | 1.00 (reference) | 1.00 (reference) |
| Quartile 2 | 1.31 (0.94-1.81) | 1.37 (0.98-1.91) |
| Quartile 3 | 1.41 (1.02-1.95) | 1.51 (1.08-2.11) |
| Quartile 4 | 2.02 (1.49-2.74) | 2.14 (1.55-2.95) |
| *P* for trend | <0.001 | <0.001 |
| Age |  | 0.998 (0.987-1.010) |
| Gender (female vs male) |  | 1.009 (0.778-1.310) |
| BMI |  | 0.979 (0.944-1.016) |
| Systolic BP |  | 0.999 (0.992-1.006) |
| Total cholesterol |  | 0.999 (0.996-1.002) |
| GGT |  | 1.000 (0.998-1.001) |
| eGFR |  | 1.000 (0.994-1.005) |
| Smoking amount (pack-year) |  | 1.000 (0.993-1.007) |
| Alcohol intake |  | 0.968 (0.718-1.305) |
| Physical activity |  | 0.944 (0.685-1.301) |

359 incident cases of pancreatic cancer developed between 2009 and 2013 after excluding the possibility of 1-year reverse causality.

Multivariate adjusted model was adjusted for age, gender, BMI, systolic BP, total cholesterol, GGT, eGFR, smoking amount (pack-year), alcohol intake and physical activity.

**Table S4. Hazard ratios (HRs) and 95% confidence intervals (CI) for the incidence of pancreatic cancer according to the quartile of fasting blood glucose after** **excluding the possibility of 1-year reverse causality (N=18,995)**

|  | HR (95% CI) ^*^ | |
| --- | --- | --- |
|  | Unadjusted | Multivariate adjusted model |
| **Fasting blood glucose levels** |  |  |
| Quartile 1 | 1.00 (reference) | 1.00 (reference) |
| Quartile 2 | 1.33 (0.95-1.87) | 1.40 (0.99-1.98) |
| Quartile 3 | 1.36 (0.96-1.90) | 1.47 (1.04-2.09) |
| Quartile 4 | 1.97 (1.43-2.70) | 2.08 (1.49-2.92) |
| *P* for trend | <0.001 | <0.001 |
| Age |  | 1.000 (0.988-1.012) |
| Gender (female vs male) |  | 1.013 (0.771-1.330) |
| BMI |  | 0.985 (0.949-1.024) |
| Systolic BP |  | 0.999 (0.992-1.006) |
| Total cholesterol |  | 0.999 (0.996-1.002) |
| GGT |  | 1.000 (0.999-1.001) |
| eGFR |  | 1.000 (0.994-1.006) |
| Smoking amount (pack-year) |  | 0.999 (0.993-1.006) |
| Alcohol intake |  | 0.956 (0.698-1.311) |
| Physical activity |  | 0.970 (0.695-1.354) |

326 incident cases of pancreatic cancer developed between 2009 and 2013 after excluding the possibility of 1-year reverse causality.

Multivariate adjusted model was adjusted for age, gender, BMI, systolic BP, total cholesterol, GGT, eGFR, smoking amount (pack-year), alcohol intake and physical activity.
