# Supplementary material for: Fasting blood glucose and risk of incident pancreatic cancer
Source: PLoS One. 2022 Oct 27;17(10):e0274195. doi: 10.1371/journal.pone.0274195 (PMC9612540; doi:10.1371/journal.pone.0274195)
Supplement: Attachment — Submitted filename: Response to reviewers and editors.docx [file pone.0274195.s008.docx]

**Response to reviewers and editors’ request**

**TITLE: Fasting blood glucose and risk of incident pancreatic cancer**

Additional Editor Comments:

Please make sure to include all the additional figures (example: Kaplan-Meir plot,..) or tables (example: Table referred under Reviewer 2's comment#1) in the manuscript or the supplemental data.

🡪 We are very appreciating to editor. As the suggestion by the editor, we included all additional figures or tables made during the review process in the manuscript or supplementary material. Table referred under Reviewer 2's comment#1 is shown in Table S2 and Kaplan-Meir plot is shown in Supplementary Figure S2, and an explanation of supplementary tables and figure is described in the manuscript.

The lower sentence is a newly added sentence actually described in the results section.

Line 237-245: The association between fasting blood glucose level and pancreatic cancer was also evaluated using a Kaplan-Meier analysis (Supplementary Figure S1). After adding triglyceride to covariates, the hazard ratios and 95% confidence interval for incident pancreatic cancer comparing the quartile 2,3 and 4 *vs* quartile 1 (reference group) were 1.45 (1.01-2.08), 1.55 (1.08-2.23) and 2.37 (1.72-3.27) (Supplementary Table S2). The adjusted hazard ratios increased in the quartiles 2 and 3 of the models that excluded people who developed pancreatic cancer within six months or a year of entering the cohort (Supplementary Table S4, Supplementary Table S5).

Supplementary Figure S1. Cumulative incidence for the development of pancreatic cancer

Table S2. Hazard ratios (HRs) and 95% confidence intervals (CI) for the incidence of pancreatic cancer according to the categories of fasting blood glucose

|  | HR (95% CI) ^*^ | |
| --- | --- | --- |
|  | Unadjusted | Multivariate adjusted model |
| **Fasting blood glucose levels** |  |  |
| Quartile 1 | 1.00 (reference) | 1.00 (reference) |
| Quartile 2 | 1.39 (1.01-1.92) | 1.45 (1.01-2.08) |
| Quartile 3 | 1.50 (1.09-2.07) | 1.55 (1.08-2.23) |
| Quartile 4 | 2.18 (1.62-2.95) | 2.37 (1.72-3.27) |
| *P* for trend | <0.001 | <0.001 |
| Age |  | 0.996 (0.985-1.008) |
| Gender (female vs male) |  | 0.964 (0.752-1.237) |
| BMI |  | 0.981 (0.947-1.017) |
| Systolic BP |  | 0.999 (0.992-1.006) |
| Triglyceride |  | 1.000 (0.999-1.001) |
| GGT |  | 1.000 (0.999-1.001) |
| eGFR |  | 1.001 (0.995-1.006) |
| Smoking amount (pack-year) |  | 0.999 (0.993-1.006) |
| Alcohol intake |  | 0.974 (0.730-1.299) |
| Physical activity |  | 0.946 (0.694-1.291) |

Multivariate adjusted model was adjusted for age, gender, BMI, systolic BP, triglyceride, GGT, eGFR, smoking amount (pack-year), alcohol intake and physical activity.
